# Citalopram Administration Does Not Promote Function or Histological Recovery after Spinal Cord Injury

**DOI:** 10.3390/ijms21145062

**Published:** 2020-07-17

**Authors:** Rui Lima, Susana Monteiro, Eduardo D. Gomes, Natália L. Vasconcelos, Rita Assunção-Silva, Mónica Morais, António J. Salgado, Nuno A. Silva

**Affiliations:** 1 Health Sciences Research Institute (ICVS), School of Medicine, University of Minho, Campus de Gualtar, 4710-057 Braga, Portugal; id6527@alunos.uminho.pt (R.L.); susanamonteiro@med.uminho.pt (S.M.); eduardogomes@med.uminho.pt (E.D.G.); id8272@alunos.uminho.pt (N.L.V.); rcribeirosilva@gmail.com (R.A.-S.); monica.dias-morais@pasteur.fr (M.M.); asalgado@med.uminho.pt (A.J.S.); 2 ICVS/3B’s–PT Government Associate Laboratory, University of Minho, 4710-057/4805-017 Braga/Guimarães, Portugal

**Keywords:** spinal cord injury, citalopram, SSRI, motor recovery, 5-HT

## Abstract

Citalopram is a selective serotonin reuptake inhibitor, and although widely used as an antidepressant, this drug has also demonstrated interesting repairing properties leading to motor recovery and pathology amelioration in animal models of stroke and degeneration. Here, we tested the efficacy of both 7-day and 8-week citalopram treatment in a contusive spinal cord injury (SCI) rat model. A combination of behavioral tests, histological and serum cytokine analysis was used to assess overall recovery. Despite promoting a mild reduction of inflammatory cells as well as an early, but transient increase of specific serum cytokines, citalopram administration showed no overall beneficial effects on motor performance or lesion extension. Our results do not support citalopram treatment as a therapeutic strategy for SCI.

## 1. Introduction

Spinal cord injury (SCI) leads to a broad spectrum of permanent neurological deficits and currently, there is no fully effective treatment available. Damage to motor tracts leads to severe impairments on locomotor function and depending on the anatomic level of the injury it can lead to tetraplegia or paraplegia.

The serotonergic system is important for locomotion and it has been shown to be involved in crucial aspects of SCI recovery [1,2]. Serotonin (5-HT) activates central pattern generators in both invertebrate and vertebrate organisms [3,4] and 5-HT or agonist administration improves locomotion after SCI [2]. Serotonergic neurotransmission is associated with axonal plasticity and regeneration. In the intact central nervous system (CNS), axonal 5-HT fibers sprout in response to increases in 5-HT neurotransmission, and after injury, rostral (but not caudal) 5-HT fibers are still able to sprout [5,6], most likely due to the preservation of inputs from brain stem 5-HT. Therefore, strategies to boost serotonin levels after SCI may be an interesting therapeutic approach to improve regeneration and functional recovery.

Selective Serotonin Reuptake Inhibitors (SSRI) are commonly used as antidepressants. They act by blocking a serotonin transporter, SERT, inhibiting 5-HT reuptake and therefore increasing serotonin extracellular levels and serotonergic neurotransmission. In addition to the modulation of serotoninergic neurotransmission and the impact on regenerating axons, treatment with SSRI has also demonstrated the ability to support an important mechanism for neuronal regeneration—increasing the generation of new neurons. Indeed, a central challenge for CNS repair is the lack of replacement of neuronal loss with new neurons in order to reestablish synaptic connections and achieve functional recovery. Contrarily to some invertebrates, mammals exhibit reduced neurogenic activity in the adult spinal cord [5,6] and therefore strategies to boost regenerative cell plasticity are needed.

Citalopram is an example of an SSRI that is well tolerated by depressed patients and therefore is commonly used in the clinical psychiatric setting [7]. Furthermore, this drug has been shown to act in the spontaneous process of cytogenesis by increasing numbers of neuroblasts [8,9], promoting neuronal differentiation [10], or even by correcting aberrant neurogenesis [11]. Over the last years, a growing body of evidence suggests that citalopram also shows promise as treatment of other diseases. In a model of ischemic stroke, citalopram-treated animals had improved sensorimotor recovery when compared to vehicle-treated ones [12]. Interestingly, this recovery was correlated with increased numbers of proliferating progenitor cells in the subventricular zone and neuroblast migration to the peri-infarcted zone [12].

In a model of Machado Joseph-disease—The CMVMJD135 mouse, chronic treatment with citalopram also promoted remarkable results rescuing motor function and ameliorating histopathologic hallmarks of the disease [13].

Taking into consideration the above mentioned references, we hypothesized that citalopram may enhance the formation of new neurons that will help rewire the injured spinal cord. Therefore, in this work, we aimed to test the therapeutic efficacy of either 7-day or 8-week administration of citalopram after a contusion SCI. Locomotor recovery was evaluated during a 8-week period, followed by an analysis of neurogenesis, inflammatory response, neuronal survival, lesion size and astrogliosis in order to evaluate citalopram’s efficacy.

## 2. Results

### 2.1. Seven-Day Citalopram Treatment Promotes a Mild and Transient Increase in the Levels of Circulating IL-1β and IL-4 24 h after SCI

**Figure 1:**
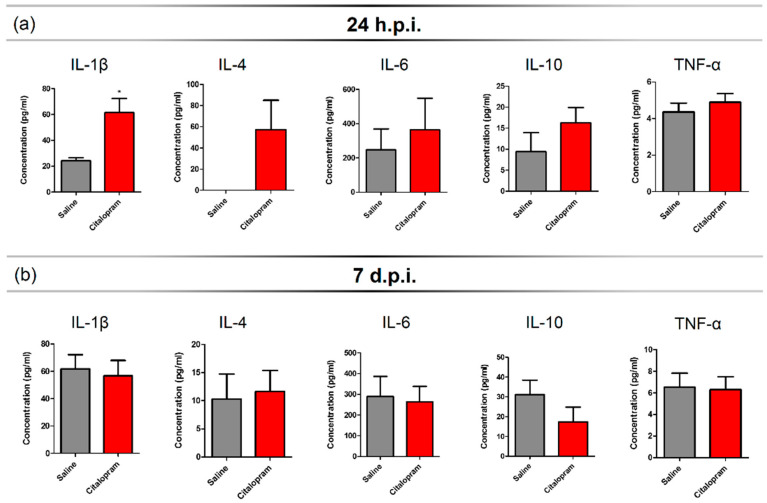
Serum cytokine profile after 7-day citalopram treatment. Levels of IL1β, IL-4, IL-6, IL-10 and TNF-α were measured in the serum collected at (**a**) 24 h post-injury (hpi) and (**b**) 7 days post-injury (dpi) from animals treated with citalopram (*n* = 5) or vehicle (*n* = 5). Values shown as mean ± SEM. * *p* < 0.05.

An SCI triggers a strong inflammatory response that recruits peripheral immune cells that infiltrate the lesioned spinal cord. In order to assess the impact of 7-day citalopram (Lundbeck, Copenhagen Denmark) treatment (intraperitoneal [i.p.] injection) on the levels of circulating cytokines, we collected blood samples at 24 h post-injury (hpi) and 7 days post-injury (dpi). Multiplex analysis was performed for a panel of pro- and anti-inflammatory cytokines. We observed that injured animals had no detectable serum levels of interleukin (IL)-4—A cytokine associated with a pro-regenerative phenotype of myeloid cells, 24 h after the lesion (Figure 1a). However, in the citalopram-treated animals, the proinflammatory cytokine IL-1β (*t*(8) = 3.364, *p* = 0.0099) was significantly increased when compared to the vehicle group (Figure 1a). Additionally, no differences were found between groups regarding the levels of IL-6, tumor necrosis factor (TNF)-α and interferon (IFN)-γ at this time-point (Figure 1a).

At 7-dpi, the levels of IL-1β and IL-4 of citalopram-treated animals returned to similar levels to those of vehicle-treated animals (Figure 1b) and overall, no differences were observed in any of the analyzed cytokines (Figure 1b). All statistical analysis results (positive and negative) can be found on Table S1 data.

### 2.2. Seven-Day Citalopram Treatment Decreases the Density of Microglia/Macrophages Caudally to the Lesion

**Figure 2:**
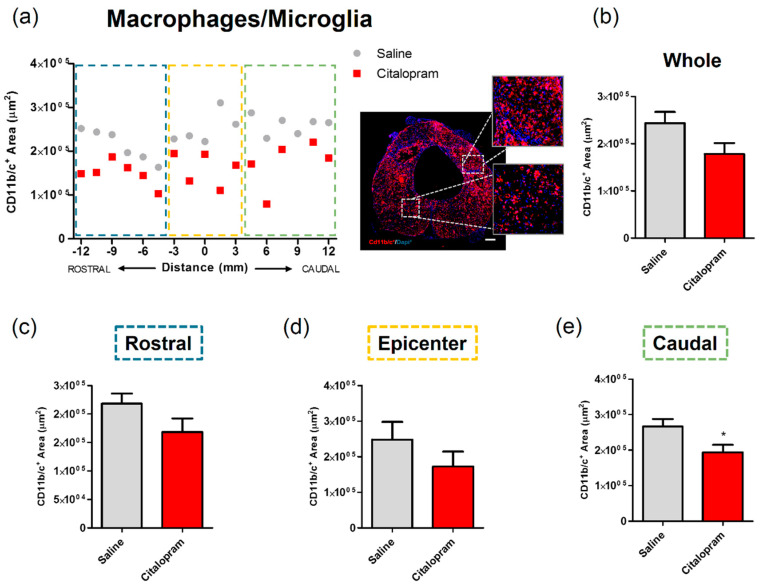
Impact of 7-day citalopram treatment on macrophages/microglia. (**a**) Distribution of CD11b/c+ cell area along the rostro–caudal axis of the spinal cord; (**b**) quantification of the area occupied by CD11b/c+ cells in the total analyzed area. Analysis of the spinal cord was then subdivided into (**c**) rostral, (**d**) lesion epicenter and (**e**) caudal areas of citalopram- (*n* = 5) vs. vehicle-treated animals (*n* = 6). Values shown as mean ± SEM; * *p* < 0.05. Scale bar: 200 μm.

After 8 weeks of the initial SCI, spinal cord tissue was collected for histological analysis to assess the long-term impact of the 7-day citalopram treatment. Analysis of the expression of CD11b/c—a marker for macrophages and microglia was performed to assess the impact of treatment on local inflammation. The density of macrophages/microglia present in the spinal cord was quantified by measuring the area occupied by CD11b/c-expressing cells in two different regions of the spinal cord: the injured area and the spared surrounding tissue (Figure 2). This analysis revealed that citalopram treatment did not significantly affect the area occupied by macrophages rostrally and at the epicenter region of the lesion (Figure 2c,d). However, a statistically significant reduction was observed caudally to the lesion (*t*(9) = 2.447; *p* = 0.0369) (Figure 2e).

**Figure 3:**
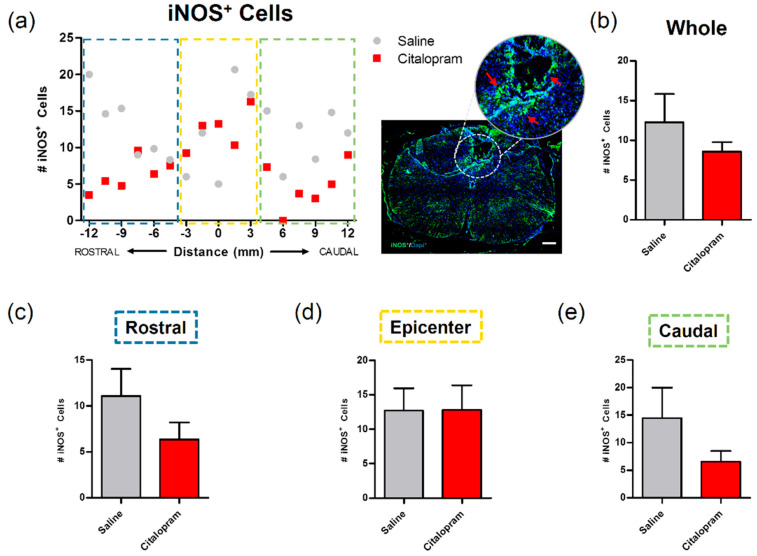
Impact of 7-day citalopram treatment on iNOS-expressing cells. (**a**) Distribution of the number of iNOS+ cells along the rostro–caudal axis of the spinal cord; (**b**) quantification of the number of iNOS+ cells in the total analyzed area. Analysis of the spinal cord was then subdivided into (**c**) rostral, (**d**) lesion epicenter and (**e**) caudal areas of citalopram- (*n* = 5) vs. vehicle-treated animals (*n* = 6). Values shown as mean ± SEM. Scale bar: 200 μm.

The expression of inducible nitric oxidase synthase (iNOS) by cells present in the spinal cord was analyzed to assess their proinflammatory profile. Macrophage iNOS expression is associated with a highly proinflammatory phenotype that contributes to further tissue damage. Reducing the proinflammatory profile of these cells—or even promoting/shifting to a pro-regenerative phenotype—can represent an interesting immunomodulatory strategy to promote neuroprotection [14,15]. Here, we tested the citalopram immunomodulatory potential by assessing the number of iNOS positive cells. However, we observed that the 7-day administration of citalopram had no significant impact on the number of iNOS positive cells in any of the analyzed areas of the spinal cord (Figure 3).

### 2.3. Seven-Day Citalopram Treatment Did Not Impact Different Neuronal Populations

**Figure 4:**
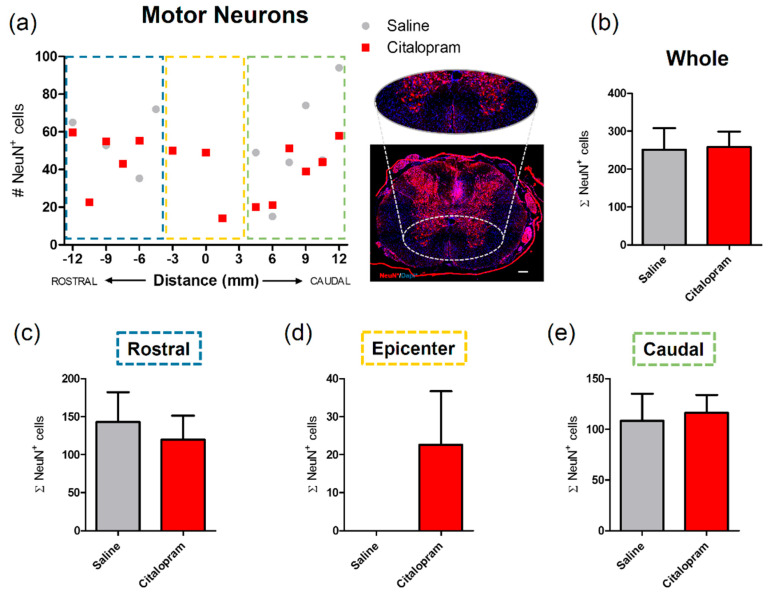
Impact of 7-day citalopram treatment on motor neurons. (**a**) Distribution of the number of NeuN+ cells in the ventral horns, along the rostro–caudal axis of the spinal cord; (**b**) quantification of NeuN+ cells in the total analyzed area. Analysis of the spinal cord was then subdivided into (**c**) rostral, (**d**) lesion epicenter and (**e**) caudal areas of citalopram- (*n* = 5) vs. vehicle-treated animals (*n* = 6). Values shown as mean ± SEM. Scale bar: 200 μm.

The effect of citalopram 7-day treatment on the protection/regeneration of neuronal populations of the spinal cord was assessed at 8 weeks post-injury (wpi). The number of motor neurons at the ventral horns was counted based on the expression of NeuN—a neuronal marker. There was no significant difference in the numbers of motor neurons after citalopram treatment in the rostral (Figure 4c) and caudal regions (Figure 4e). However, neurons were found at the lesion epicenter in some citalopram-treated animals in contrast to the total absence of these cells in the same region of vehicle-treated animals. Nevertheless, this effect was not homogeneous among citalopram-treated animals, with some of these presenting a complete loss of neurons in the epicenter similarly to vehicle-treated animals (Figure 4d). Overall, no significant differences were observed in the number of motor neurons in the citalopram- vs. vehicle-treated animals (Figure 4).

**Figure 5:**
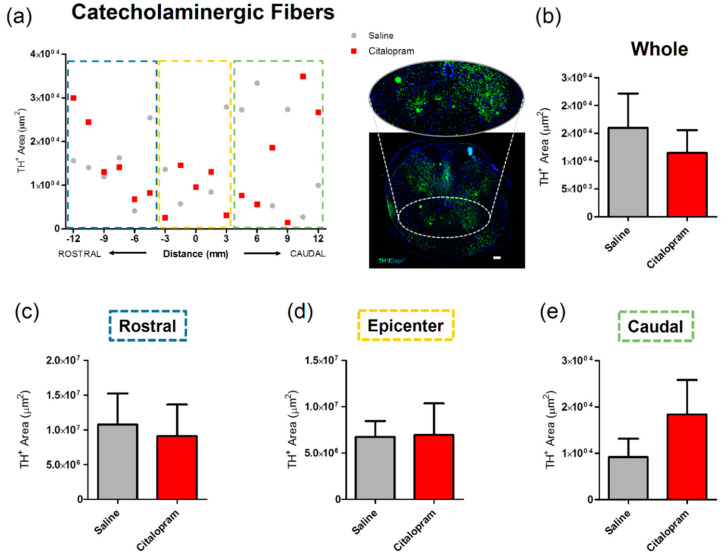
Impact of 7-day citalopram treatment on catecholaminergic fibers. (**a**) Distribution of tyrosine hydroxylase (TH)+ cell area in the ventral horns, along the rostro–caudal axis of the spinal cord; (**b**) quantification of the area occupied by TH+ cells in the total analyzed area. Analysis of the spinal cord was then subdivided into (**c**) rostral, (**d**) lesion epicenter and (**e**) caudal areas of citalopram- (*n* = 5) vs. vehicle-treated animals (*n* = 6). Values shown as mean ± SEM. Scale bar: 200 μm.

The effect of 7-day citalopram treatment was analyzed in a specific group of neurons in the spinal cord—the catecholaminergic neurons. For this purpose, the density of cells expressing tyrosine hydroxylase (TH) was quantified. Again, no major differences could be observed in any of the analyzed regions (Figure 5).

**Figure 6:**
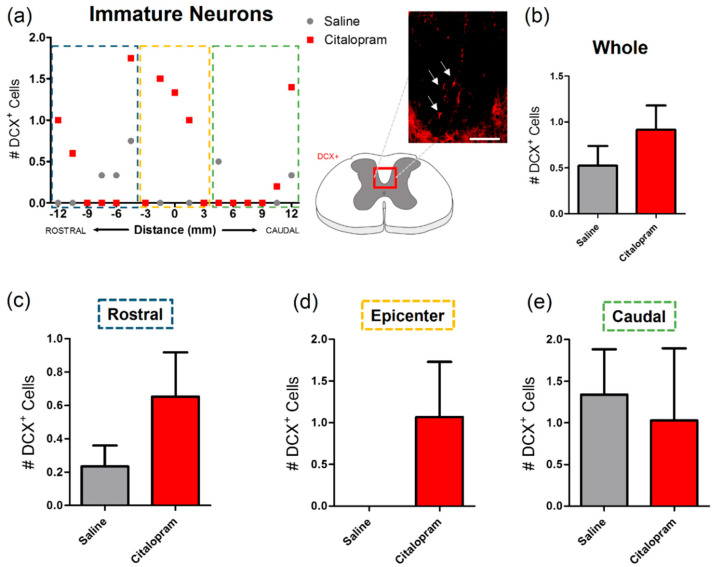
Impact of 7-day citalopram treatment on immature neurons. (**a**) Distribution of the doublecortin DCX+ cells in the corticospinal tract (white arrows), along the rostro–caudal axis of the spinal cord; (**b**) quantification of DCX+ cells in the total analyzed area. Analysis of the spinal cord was then subdivided into (**c**) rostral, (**d**) lesion epicenter and (**e**) caudal areas of citalopram- (*n* = 5) vs. vehicle-treated animals (*n* = 6). Values shown as mean ± SEM. Scale bar: 200 μm.

Since citalopram was previously described as having neurogenic potential, we focused our analysis on immature neuronal populations of the spinal cord, that are known to migrate to the corticospinal tract, based on the expression of doublecortin (DCX) (Figure 6a). Results revealed that very few cells expressed this immature neuronal marker in this region of the spinal cord (Figure 6b). No significant differences were observed in the number of DCX+ cells after citalopram treatment (Figure 6).

### 2.4. Seven-Day Citalopram Treatment Did Not Improve Functional and Histological Recovery

**Figure 7:**
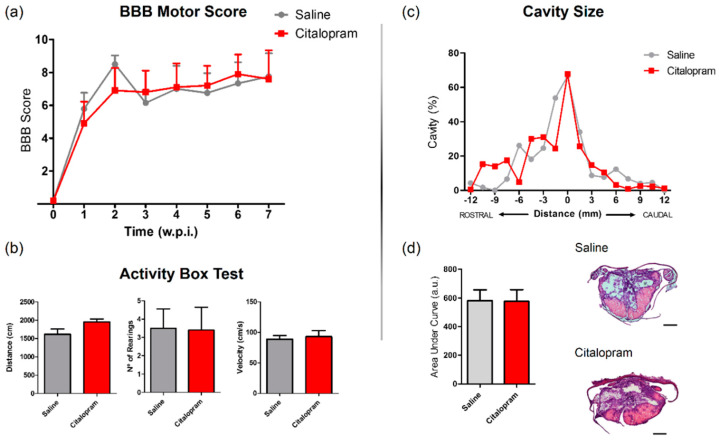
Impact of 7-day citalopram treatment on functional recovery and cavity analysis. (**a**) BBB score was used to assess motor recovery 3 days after the injury and once a week thereafter until the end of the experiment; (**b**) distance traveled, average velocity and the number of rearing behaviors was analyzed using the activity box test (ABT) to evaluate gross motor recovery of citalopram- vs. vehicle-treated animals; (**c**) analysis of the injury was performed by measurement of the cavity volume using hematoxylin–eosin staining; (**d**) analysis of cavity size considering the area under the curve. Citalopram- *n* = 7 and vehicle-treated animals *n* = 7. Values shown as mean ± SEM. Scale bar: 500 μm. BBB—Basso, Beattie and Bresnahan.

The motor behavior of the animals was analyzed once per week for a total of 8 weeks after SCI using the Basso, Beattie and Bresnahan (BBB) scale—a gold-standard method to assess motor recovery in rat SCI rat models. No statistically significant differences were observed between experimental groups regarding any of the analyzed time-points (Figure 7a).

In the last week of the experiment (8 wpi), the motor behavior was further assessed using the activity box test (ABT). Total distance traveled, average velocity the total number of rearing behaviors were assessed for 5 min. Citalopram 7-day treatment did not significantly improve any aspect of motor recovery assessed with the ABT (Figure 7b).

SCI leads to the progressive formation of a cavity in the lesion epicenter surrounded by a glial scar. Using hematoxylin–eosin staining, we measured the cavity size to assess improvements caused by citalopram treatment at the tissue level. We could not detect any reduction of the cavity volume after citalopram treatment (Figure 7c,d).

### 2.5. Eight-Week Citalopram Treatment Did Not Improve the Inflammatory Profile nor Functional and Histological Recovery

The repeated daily administration during the subacute phase of SCI pathophysiology only led to minor alterations in circulating cytokines (Figure 1) and a modest reduction of macrophages/microglia caudally to the lesion epicenter (Figure 2) while no effect of the treatment was observed regarding functional and histological correlates of recovery (Figure 7). We then hypothesized if the continuation of citalopram during the chronic stage would lead to some degree of recovery. In order to achieve that, after the 7-day treatment (i.p. injection), citalopram was continuously made available in the drinking water during 8 weeks.

**Figure 8:**
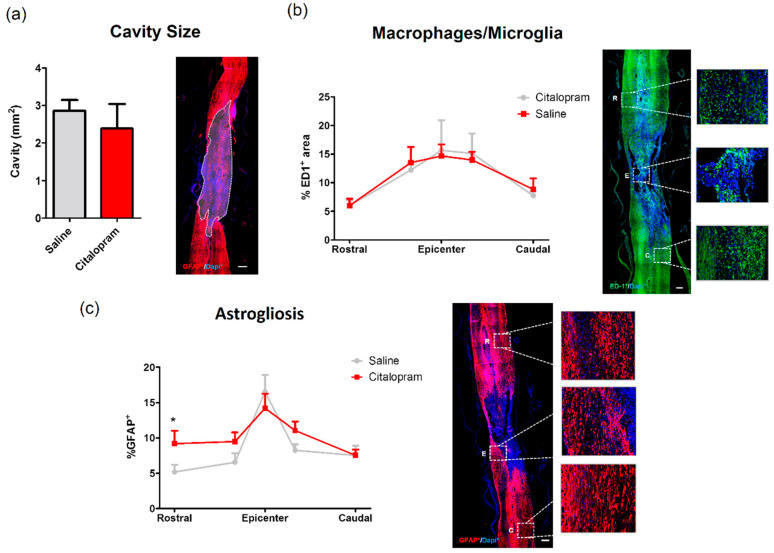
Impact of 8-week citalopram treatment on cavity size, macrophages/microglia and astrogliosis. (**a**) Analysis of the injury was performed by measurement of the% of the area occupied by the cavity using glial fibrillary acidic protein (GFAP) staining; (**b**) distribution of ED1+ cell area (macrophages/microglia) along the rostro–caudal axis of the spinal cord; (**c**) distribution of GFAP+ cell area along the rostro–caudal axis of the spinal cord. Citalopram- *n* = 7 and vehicle-treated animals *n* = 8. Values shown as mean ± SEM. * *p* < 0.05.

At the end of the experiment, we analyzed the cavity size and the density of macrophages/microglia (ED1+ cell area) present in the spinal cord, however, we found no effect of 8-week citalopram treatment in any of the analyzed regions (Figure 8a,b). The analyses of astrogliosis did not reveal differences in the epicenter and caudal region of the spinal cord, however we observed a significant increase of astrocytes rostrally to the lesion site (Figure 8c).

**Figure 9:**
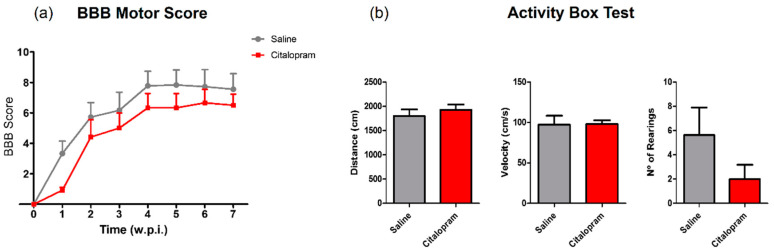
Impact of 8-week citalopram treatment on functional recovery. (**a**) BBB score was used to assess motor recovery 3 days after the injury and once per week thereafter until the end of the experiment; (**b**) distance traveled, velocity and the number of rearing behaviors were analyzed using the ABT test to evaluate gross motor recovery. Citalopram- *n* = 7 and vehicle-treated animals *n* = 8. Values shown as mean ± SEM. BBB—Basso, Beattie and Bresnahan; ABT— activity box test.

Moreover, the 8-week treatment with citalopram did not improve motor deficits assessed by the BBB and the ABT (Figure 9a,b, respectively). Moreover, it did not reduce the size of the cavity as well (Figure 8a).

## 3. Discussion

Spinal cord injury leads to devastating neurological deficits and currently there is no fully effective treatment for this condition. The development of new therapies promoting neuroprotection and regeneration after SCI has not improved significantly in the last decades. Additionally, the translation of candidate therapies with proven efficacy in preclinical testing to clinical trials endures a long process due to uncertainty regarding human safety. In this context, drug repurposing strategies, which rely on finding new uses for existing FDA approved compounds, have been gaining pace.

Here, we tested the therapeutic potential of citalopram—a serotonin reuptake inhibitor mostly known for its therapeutic use in depression, in a preclinical rat model of SCI. Citalopram is a modulator of serotonergic transmission through inhibition of serotonin reuptake, although other actions have been described, such as the promotion of the generation of new neurons [8,9] and immunomodulation [16,17]. The failure or modest spontaneous recovery observed after an SCI is associated both to the low regenerative capacity of the adult spinal cord as well as with secondary mechanisms of injury that hamper recovery and extend neurological deficits. The combination of citalopram actions in serotonergic transmission, neurogenesis, and immunomodulation—along with the fact that it is a very well tolerated drug–lead us to hypothesize whether citalopram had therapeutic efficacy in SCI.

Citalopram treatment had a marginal effect in specific serum cytokines levels at a very early time-point following the injury. After 24 hpi, it led to increased levels of IL-4, a cytokine associated with the promotion of regeneration by monocytes/macrophages, in contrast with controls where this cytokine was not detected. However, treatment also increased the proinflammatory cytokine IL-1β, demonstrating that its immunomodulatory effect is not restricted to promoting an anti-inflammatory phenotype. Indeed, this observation is in line with in vitro studies showing that stimulating blood cells with citalopram promotes an increase in specific cytokines secretion, including IL-1β [16]. After seven dpi, we observed not only an overall increase in the levels of the proinflammatory cytokines IL-1β, IL-6 and TNF-α, but also an increase in anti-inflammatory cytokines IL-10 and IL-4 when compared to the levels at 24 hpi, which most likely reflects the dynamic progression of an active inflammatory response. The modest effect of citalopram in the levels of IL-4 and IL-1β observed at 24 hpi was however lost, and a generalized increase in the levels of circulating cytokines was observed at seven dpi.

These early changes in systemic inflammatory cytokines had a mild influence on the number of inflammatory infiltrating cells in the spinal cord. In a long-term analysis, at eight weeks after the initial SCI, no major differences could be observed in the number of macrophages/microglia present rostrally or in the lesion epicenter, however, a significant decrease was observed caudally.

The early and transient effects of citalopram in inflammatory cytokines did not have a significant impact on neuroprotection following SCI with no differences observed after treatment either in the density of motor neurons nor in the catecholaminergic neuronal populations in the spinal cord.

Another interesting hypothesis that we pursued was that citalopram could boost the formation of new neurons which are important to replace dead neurons. Doublecortin expression is widely used for neurogenesis studies labeling newly generated neurons from neurogenic niches in the brain. Its expression has also been observed in non-neurogenic places, such as the piriform cortex [18] or the spinal cord meninges [19]. After SCI, DCX-cells migrate from the spinal meninges to the spinal cord parenchyma to integrate the glial scar [19], although the response of these cells to citalopram was not known. We found a residual number of DCX-expressing cells in the spinal cord after spinal cord injury. However, our data do not support the modulation of these cells by citalopram, as no effect in the number of DCX-expressing cells could be found after treatment.

In addition to non-observable effects in neuronal populations, 7-day citalopram treatment also failed to promote motor recovery and reduction in the size of the lesion. To test the hypothesis that 7-day citalopram treatment could be overlooking regeneration mechanisms that develop later in SCI pathophysiology, we extended the treatment to eight weeks. Nonetheless, in this set of treated animals, citalopram also failed to promote significant motor and histological recovery.

Although the effects of 7-day or 8-week administration of citalopram observed in our rat SCI model does not support a therapeutic action, the possibility of testing other dosages could be considered.

The absence of effect in motor recovery by citalopram treatment may support studies demonstrating that motor recovery after SCI is not due to serotonin levels, but rather to the constitutive activity of specific 5-HT receptors [20]. In fact, in a regenerating model of SCI—in lampreys, endogenous serotonin has shown to inhibit axonal regeneration of specific descending tracts [21] highlighting a dual role of serotonin for regeneration.

In conclusion, collectively our data does not support sub-acute or chronic citalopram administration as a treatment for SCI. However, given that this is a negative study it is important to point out the limitations of our work. The study mainly focused on motor and histological recovery; however, we did not analyze autonomic or sensory effects. Possible recovery of these dimensions cannot be discarded. We cannot also discard that citalopram may have a therapeutic effect on mild to moderate lesions or on different anatomic regions. For instance, with a less severe injury there is a higher likelihood of having more serotonin crossing the injury site and reaching the lumbar enlargement. In this scenario, where there is more availability of serotonin, the citalopram effect on serotonin levels may boost the activation of the central pattern generator located at the lumbar region, therefore, improving locomotion. Additionally, we administered citalopram using two dosages (8 and 10 mg/kg/day) that roughly compare to the high dosage range prescribed to human patients for depression [22] and two routes of administration. Previous work demonstrated that citalopram at this dosage has a biologic active role on the brain and spinal cord tissue [12,13]. Espinera et al. performed i.p. injections of citalopram and observed a therapeutic action on ischemic stroke induced mice [12]. On the other hand, Teixeira-Castro et al. administered citalopram in the drinking water of animal models of Machado–Joseph disease and also observed a therapeutic effect [13]. We used both routes of administration and in none of them obtained positive results. Spinal cord injury can influence drug absorption, however, as we are using a high dosage, we do not believe it would be significant to exclude citalopram from having a therapeutic effect. That being said, we cannot rule out that a different dosage may be effective. Finally, we only used female rats because they have a higher survival rate than males when subjected to this type of injury. Sex is a biologic variable that may play a role in our study and so we cannot dismiss a therapeutic effect of citalopram on male SCI animal models. Moreover, it is also possible that by increasing the power of our sample size the differences between groups could become significant.

## 4. Materials and Methods

### 4.1. Animals

Twenty-six Female Wistar rats (Charles River, USA), 14 weeks old, weighing 210–260 g were maintained at the animal facilities of the Institute of Life and Health Sciences (ICVS, Braga, Portugal) under standard laboratory conditions (12 h light: 12 h dark cycles, 22 °C, relative humidity of 55%, ad libitum access to standard food and water) and housed in pairs. All procedures were carried out following the European Union Directive 2010/63/EU and were approved by the ethical committee in life and health sciences (ID: SECVS116/2016, University of Minho, Braga, Portugal). Animals were handled daily for 3 consecutive days before surgery for habituation and stress reduction.

### 4.2. Spinal Cord Injury Model and Treatment

A weight drop trauma model was used to induce a severe contusion injury as previously described [14,23,24]. General anesthesia was induced by i.p. injection (of ketamine (100 mg/mL, Imalgene/Merial, Duluth, GA, USA) and medetomidine hydrochloride (1 mg/mL, Dormitor/Pfizer, New York, NY, USA) mixture, at a volume ratio of 1.5:1. Once anesthetized, animals received subcutaneous injections of the analgesic butorphanol (10 mg/mL, Butomidor/Richter Pharma AG, Wels, Austria), and the antibiotic enrofloxacin (5 mg/mL, Baytril/Bayer, Leverkusen, Germany). The fur was shaved from the surgical site and the skin disinfected with ethanol 70% and chlorohexidine. Surgical procedures were performed under sterile conditions. The animals were placed in a prone position and a dorsal midline incision was made at the level of the thoracic spine (T5–T12). The paravertebral muscles were retracted and the spinous processes and laminar arc of T8 were removed, and the spinal cord exposed. The dura was left intact. A weight drop trauma model was used, that consisted of dropping a 10 g weight rod from a 20 cm height onto the exposed spinal cord [14,23,24]. The rod was guided through a stabilized tube that was positioned perpendicularly to the center of the spinal cord. After the trauma, the muscles were sutured with Vicryl suture 4–0 (Johnson and Johnson, New Brunswick, NJ, USA) and the incision closed with surgical staples (Fine Science Tools, Heidelberg, Germany). Anesthesia was reversed using atipamezole (5 mg/mL, Antisedan/Pfizer, New York, NY, USA).

**Figure 10:**
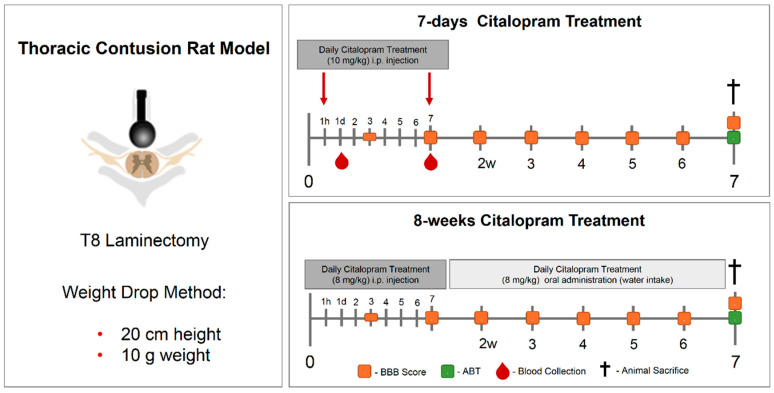
Experimental layout of the in vivo experiment testing the therapeutic efficacy of 7-day and 8-week citalopram treatment. A thoracic (T8) contusive SCI rat model was used. The 7-day treatment with citalopram was administered one hour after the injury and then repeated once daily for 7 days. The 8-week citalopram treatment was performed in the first week as the 7-day treatment, and then citalopram was continuously provided in the drinking water until the end of the experiment. Blood was collected from the tail on day 1 and 7 post-injury for cytokine analysis. Motor recovery was analyzed using the BBB score on day 3 after the injury and then once per week until the end of the experiment. ABT was performed 7 weeks post-injury and the animals sacrificed. SCI—spinal cord injury; BBB—Basso, Beattie and Bresnahan; ABT—activity box test.

A 7-day treatment approach starting one-hour post-injury was performed (Figure 10).Animals were randomly divided into two experimental groups: citalopram (*n* = 5, citalopram hydrobromide 10 mg/kg (Lundbeck, Copenhagen, Denmark)) or controls receiving vehicle (*n* = 6, saline). In the 7-day set, treatment was administered daily via i.p. injection for 7 days. For the 8-week therapeutic approach, animals were randomly divided into citalopram (*n* = 7, 8 mg/kg/day) and vehicle (*n* = 8). The 8-week treatment was administered by i.p. injection in the first week, and the following weeks until sacrifice (8 wpi) in the drinking water. For this both the rats’ water intake [25] and citalopram half-life time were taken into account, being renewed every 72 h.

Postoperative care included butorphanol (Richter Pharma AG, Wels, Austria) administration twice per day, for 5 days as well as vitamins (Duphalyte, Pfizer, New York, NY, USA), saline and enrofloxacin (Bayer, Leverkusen, Germany), twice per day for 7 days. Manual expression of bladders was performed twice per day until animals recovered spontaneous voiding. Bodyweight was monitored weekly as a parameter of the overall health of the animals. In the event of a weight loss of over 10% of body weight, a high-calorie oral supplement (Nutri-Cal^®^, Ventoquinol, Fort Worth, TX, USA) was administered daily.

### 4.3. Behavioral Assessment

#### 4.3.1. Basso, Beattie and Bresnahan (BBB)

The Basso, Beattie and Bresnahan (BBB) locomotor rating scale [26] was used to evaluate functional recovery. Researchers performed all behavioral tests blindly to the treatment group. The BBB test was performed three days post-injury and thereafter once per week until the end of the experiment. A BBB score of 0 indicates no hindlimb movement. A BBB score of 1 through 8 indicates joint movement, but no weight support. A BBB score of 9 through 20 indicates an ability to support weight and use the limb for locomotion, but with some degree of abnormality. A BBB score of 21 corresponds to a normal rat’s locomotion.

#### 4.3.2. Activity Box Test (ABT)

The activity box test was used to assess gross motor behavior. The analysis included the total distance traveled, average velocity and the number of rearings. Animals were allowed to freely explore an open arena (43.2 cm × 43.2 cm) with transparent acrylic walls (Med Associates, Inc., St Albans, VT, USA) for a total time of 5 min. Data were collected automatically by infrared beam detection using the activity monitor software (4v, Med Associates, Inc., St Albans, VT, USA).

### 4.4. Cytokine Analysis

After 24 h and 7 dpi (Figure 10), blood was collected from the tail and allowed to clot for 30 min before centrifugation (10 min at 10,000× *g*). Then, serum was collected and frozen at −80 °C. Cytokine quantification was performed using a multiplex assay for cytokine magnetic bead panel (MILLIPLEX^®^ MAP kit) for interleukin (IL) 1β, IL-4, IL-6, IL-10 and tumor necrosis factor (TNF)-α detection (Millipore) as instructed by the supplier. A standard control (0 ng/mL) containing only the assay buffer was used for removing the background signal. Moreover, analyte quantification for two different quality controls present in the multiplex kit was performed. Both quality controls were within the range provided by the manufacturer. Samples were analyzed in a MAGPIX Luminex’s xMAP^®^ instrument (Luminex, Austin, TX, USA). Analyte concentrations in samples were calculated through median fluorescent intensity (MFI) of standard controls using a 5-parameter logistic curve-fitting method.

### 4.5. Histological Assessment

Eight-weeks post-injury, animals were deeply anesthetized by an i.p. injection of sodium pentobarbital (200 mg/mL, Eutasil/Ceva Sante Animale, Libourne, France) and transcardially perfused with 100 mL of cold 0.9% saline followed by 300 mL of 4% paraformaldehyde (PFA) in 1× phosphate-buffered saline (PBS). A rough dissection of the vertebral column and spinal cord was performed, and tissues were fixed in a solution of 4% PFA for 24 h (4 °C). The spinal cord was then dissected from the vertebral column and immersed in a cryoprotectant solution—30% sucrose, for 48 h at 4 °C. Afterward, 2 cm length of spinal cord tissues, centered on the lesion, were submerged in optimal cutting temperature (OCT) embedding medium, frozen on dry ice and stored at −20 °C. To minimize bias, each spinal cord was coded to keep the experimenter blind to the treatment.

Cross-sections (for the 7-day treatment animal set) and longitudinal sections (for the 8-week treatment animal set) of 20-μm thickness were performed using a cryostat (Leica CM1900, Leica Biosystems, Nussloch, Germany) and thaw-mounted onto charged microscope slides (Superfrost Plus, Thermo Scientific, Waltham, MA, USA). Histological preparation and analysis were performed blindly to the treatment group.

#### 4.5.1. Lesion Size Analysis

Tissue cross-sections from the 7-day treatment set were stained for hematoxylin–eosin staining and then photographed with a stereology microscope (Zeiss Axioplan 2 Imaging, Jena, Germany) using a 2.5× objective. Longitudinal tissue sections from the 8-week treatment set were immunofluorescence stained against glial fibrillary acidic protein and then photographed using a confocal point-scanning microscope, Olympus FV1000. Evaluation of damaged tissue and cavity area was performed on cross-sections (150 μm apart) along the rostro–caudal axis and longitudinal sections (200 μm apart) along the dorsal-ventral axis. The areas were manually traced and quantified using ImageJ software (National Institutes of Health, Bethesda, MD, USA).

#### 4.5.2. Immunohistochemistry Protocol

For immunofluorescence staining, slices were washed with PBS, permeabilized with 0.2% Triton X-100 for 10 m, and blocked with 5% fetal calf serum in 0.2% Triton X-100 for 30 m. Afterward, the following primary antibodies were incubated overnight at room temperature (RT): mouse anti-CD11b/c for microglia/macrophages (1:100; Pharmingen, San Diego, CA, USA); rabbit inducible nitric oxide synthase (iNOS) for activated microglia/macrophages (1:100; Millipore, Darmstadt, Germany), rabbit anti-tyrosine hydroxylase (TH) (AB152) for catecholaminergic neurons (1:1000; Millipore, Darmstadt, Germany), rabbit anti-glial fibrillary acidic protein (GFAP) for astrocytes (1:200; Dako Denmark, Glostrup, Denmark), guinea pig anti-doublecortin (DCX) (AB2253) for new born neurons (1:300; Millipore, Darmstadt, Germany), mouse anti-macrophages/monocytes clone ED-1 (1:300, Millipore, Darmstadt, Germany) and mouse anti-NeuN for motor neurons (1:200, Millipore, Darmstadt, German). The following day primary antibodies were then probed (2 h incubation) with the appropriate Alexa 594- or Alexa 488-conjugated secondary antibodies (1:1000; Invitrogen, Paisley, UK). Sections were counterstained with 4′,6-diamidino-2-phenylindole (DAPI) for 30 min (1:1000; Sigma, Saint Louis, CA, USA) and mounted with Immu-Mount^®^ (Thermo Scientific, Waltham, MA, USA). Between steps, 5 washes with PBS were performed. For all immunofluorescence procedures, the appropriate negative controls were obtained by the omission of the relevant primary antibody. Images were acquired using a confocal point-scanning microscope, Olympus FV1000. All images were analyzed using ImageJ and FIJI software (1.47v, NHI, Bethesda, Maryland, USA). The negative controls were performed by omitting the primary antibody on the immunohistochemistry protocol (Figure S1).

#### 4.5.3. Immunofluorescence Analysis

For the 7-day-spinal cord animal set tissue photomicrographs were collected every 150 μm both rostrally and caudally from the epicenter. The epicenter region was considered the area ranging from −300 μm and 300 μm surrounding the lesion epicenter. The most rostral area analyzed extended from −1200 μm to −300 μm from the lesion epicenter and the most caudal area analyzed extended from 300 μm to 1200 μm from the lesion epicenter. Photomicrographs were collected from the 8-week-treatment animal set tissue at every 200 μm from the dorsal to the ventral side of the spinal cord.

The immunofluorescence quantification in each photomicrograph was assessed by positive-cell counting (for the iNOS, NeuN and DCX) or positive staining area (for the CD11b/c, TH, ED-1 and GFAP area). The epicenter (section with the largest cavity) was at first identified, and then the analysis was done at every 150 µm rostral and caudal to the epicenter. Specifically, the analysis for the CD11b/c marker was performed in two random fields of each photomicrograph. Since the staining for this marker in the epicenter region was heterogeneous due to the presence of cavitation, the strategy was to select one field within the cavitation and other fields outside the cavitation. Quantification of iNOS+ cells was assessed in niches of positive cells in each section. NeuN+ cells were counted in the ventral horns of the gray matter. TH+ area was measured in the ventral horns of the gray matter. DCX+ cells were counted in the CST. ED-1+ and GFAP+ area on longitudinal sections were measured and quantified in five different regions (rostral, rostral-epicenter, epicenter, epicenter–caudal and caudal). Finally, the GFAP+ area was measured on the entire spinal cord slice using lower magnification. Due to cavitation, the GFAP+ area is presented in percentage for the total spinal cord tissue.

A section’s exclusion criteria for analysis were shattered, cracked or folded sections or sections washed off during the immunostaining procedure. After obtaining micrographs through confocal microscopy, the photos were opened with the Image J software. For cell counting, the multi-point tool was used. For positive area measurements, first, the scale was determined and then the images were converted to 8 bits and were processed in the menu “make binary”. Finally, using the menu “analyze particles”, the software automatically calculated the areas occupied by each marker, using the dark background as a contrast. The identification of tracts was according to the rat spinal cord atlas [27]. Data plotted in the graphs represent mean numbers (or area) per section.

### 4.6. Statistical Analysis

Statistical analysis was performed using GraphPad Prism software (6.00v, San Diego, CA, USA). The normality of the data were evaluated by the Kolmogorov–Smirnov normality tests. When the equal variances criterion was not met, Welch’s correction was applied. Data from BBB test was assessed by a repeated-measures ANOVA test. Differences between groups were compared with the post hoc Bonferroni test. Immunofluorescence and cytokine concentration data were analyzed using the Student’s t-test or Mann–Whitney according to normality results. Statistical significance was defined for *p* < 0.05 (95% confidence level). Data are shown as mean ± standard error (SEM). All statistical analysis results (positive and negative) can be found on Table S1 data.

## Supplementary Materials

Supplementary materials can be found at https://www.mdpi.com/1422-0067/21/14/5062/s1.

## Abbreviations

5-HT	serotonin
ABT	activity box test
BBB	Basso, Beattie and Bresnahan
CNS	central nervous system
DAPI	4′,6-diamidino-2-phenylindole
DCX	doublecortin
dpi	days post injury
GFAP	glial fibrillary acidic protein
hpi	hours post injury
IL	interleukin
IFN	interferon
iNOS	inducible nitric oxide synthase
ip	intraperitoneal
MST	motor swimming test
OCT	optimal cutting temperature
PBS	phosphate buffer solution
PFA	paraformaldehyde
RT	room temperature
SCI	spinal cord injury
SEM	standard error of the mean
SERT	serotonin transporter
SSRI	selective serotonin reuptake inhibitor
TH	tyrosine hydroxylase
TNF	tumor necrosis factor
wpi	weeks post-injury
