# Supplementary material for: Citalopram Administration Does Not Promote Function or Histological Recovery after Spinal Cord Injury
Source: Int J Mol Sci. 2020 Jul 17;21(14):5062. doi: 10.3390/ijms21145062 (PMC7404289; doi:10.3390/ijms21145062)
Supplement: Supplementary file 1 [file ijms-21-05062-s001.pdf]

International Journal of Molecular Sciences

MDPI

Supplementary Materials

Table S1. Statistical analysis.

Timepoint/ Animal Set	Assessment		Statistic Report
24 hpi	Cytokines	IL-1β	$t(8) = 3.364$, $p = 0.0099$
		IL-4	U = 0.0, p = 0,0168
		IL-6	$t(6) = 0.4430$, $p = 0.6733$
		IL-10	$t(9) = 1.158$, $p = 0.2767$
		TNF-α	$t(8) = 0.7574$, $p = 0.4705$
8 wpi		IL-1β	$t(9) = 0.3359$, $p = 0.7446$
		IL-4	$t(5) = 0.2325$, $p = 0.8253$
		IL-6	$t(8) = 0.2019$, $p = 0.8450$
		IL-10	$t(8) = 1.310$, $p = 0.2266$
		TNF-α	$t(9) = 0.1233$, $p = 0.9045$
7-days	Macrophages/Microglia	Whole	$t(9) = 1.940$, $p = 0.0843$
		Rostral	$t(9) = 1.729$, $p = 0.1179$
		Epicenter	$t(8) = 1.166$, $p = 0.2772$
		Caudal	$t(9) = 2.447$, $p = 0.0369$
	iNOS+ Cells	Whole	$t(9) = 0.9095$, $p = 0.3868$
		Rostral	$t(9) = 1.291$, $p = 0.2289$
		Epicenter	$t(8) = 0.02147$, $p = 0.9834$
		Caudal	$t(9) = 1.258$, $p = 0.2402$
	Motor Neurons	Whole	$t(8) = 0.1033$, $p = 0.9203$
		Rostral	$t(8) = 0.4593$, $p = 0.6582$
		Epicenter	U = 7.500, $p = 0.1797$
		Caudal	$t(8) = 0.2455$, $p = 0.8122$
	Catecholaminergic Fibers	Whole	$t(9) = 0.5735$, $p = 0.5735$
		Rostral	$t(9) = 0.2586$, $p = 0.8018$
		Epicenter	$t(7) = 0.04882$, $p = 0.9624$
		Caudal	$t(8) = 1.099$, $p = 0.3037$
	Immature Neurons	Whole	$t(9) = 1.167$, $p = 0.2733$
		Rostral	$t(9) = 1.515$, $p = 0.1642$
		Epicenter	$t(8) = 1.612$, $p = 0.1457$
		Caudal	$t(9) = 0.3131$, $p = 0.7613$
	BBB Motor Score		$F(7, 63) = 0.26$, $p = 0.9676$
	Activity Box Test	Distance	$t(9) = 1.921$, $p = 0.0869$
		Velocity	$t(10) = 0.3910$, $p = 0.7040$
		Nº of Rearings	$t(9) = 0.06159$, $p = 0.9522$
	Cavity Size		$t(9) = 0.04017$, $p = 0.9688$
8-weeks	Cavity Size		$t(11) = 0.7887$, $p = 0.4470$
	Macrophages/Microglia	Rostral	$t(11) = 0.00461$, $p = 0.9964$
		Epicenter	$t(9) = 0.1915$, $p = 0.8524$
		Caudal	$t(10) = 0.4517$, $p = 0.6611$
	Astrogliosis	Rostral	$t(14) = 2.029$, $p = 0.0620$
		Epicenter	$t(13) = 0.6756$, $p = 0.5111$
		Caudal	$t(12) = 0.00822$, $p = 0.9936$
	BBB Motor Score		$F(7, 91) = 0.91$, $p = 0.5006$
	Activity Box Test	Distance	$t(13) = 0.7122$, $p = 0.4889$
		Velocity	$t(13) = 0.04928$, $p = 0.9614$
		Nº of Rearings	$t(13) = 1.354$, $p = 0.1988$

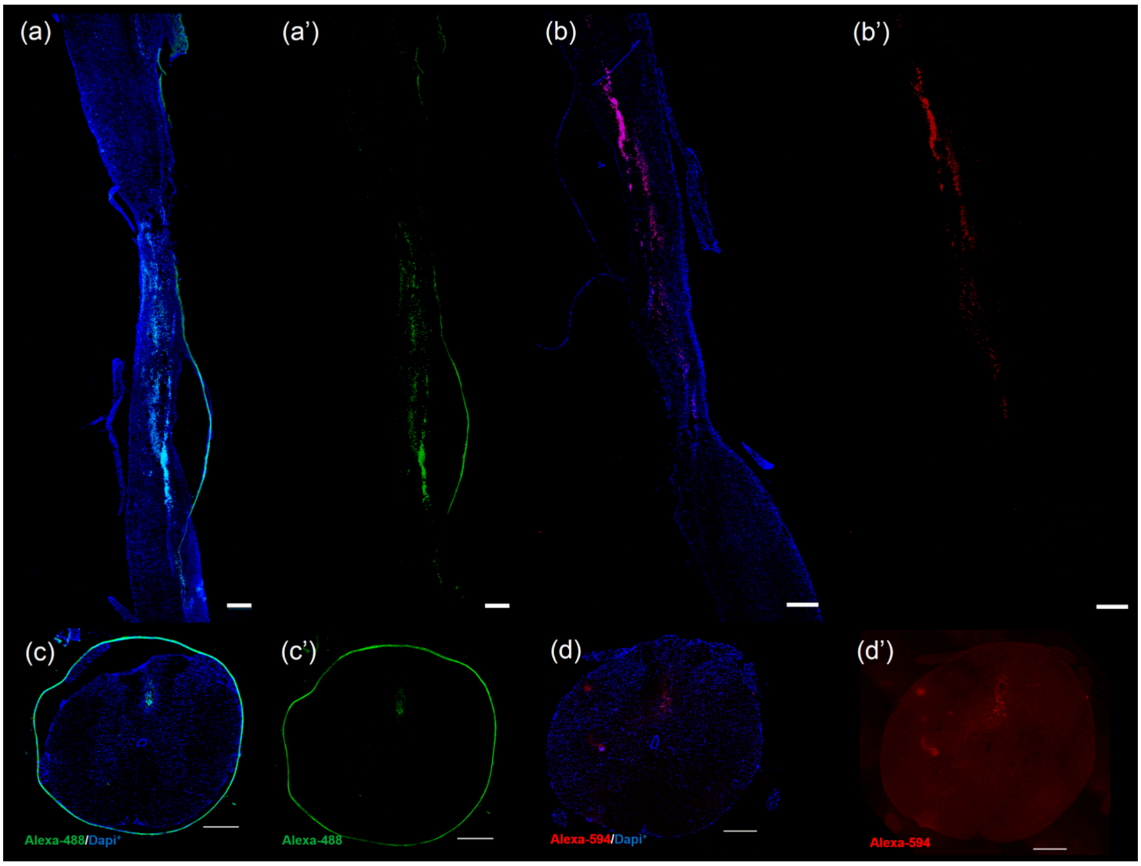

Figure S1. Negative control for immunohistochemistry analysis. Secondary antibodies Alexa-488 and Alexa-594 were incubated with spinal cord slices in the absence of the primary antibody. DAPI was used to show the general structure of the spinal cord. Unspecific staining was residual and associated with the cystic cavity. Alexa-488 unspecific staining on longitudinal and transversal spinal cord slices with (**a,c**) or without DAPI (**a′**, **c′**). Alexa-594 unspecific staining longitudinal and transversal spinal cord slices with (**b,d**) or without DAPI (**b′,d′**). Scale bar: 500 µm.

© 2020 by the authors. Submitted for possible open access publication under the terms and conditions of the Creative Commons Attribution (CC BY) license (http://creativecommons.org/licenses/by/4.0/).
